# Postmenopausal Bilateral Oophorectomy Is Associated With an Increased Risk of Restless Leg Syndrome

**DOI:** 10.1093/geroni/igaa057.733

**Published:** 2020-12-16

**Authors:** Nan Huo, Carin Smith, Liliana Gazzuola Rocca, Michelle Mielke, Walter A Rocca

**Affiliations:** Mayo Clinic, Rochester, Minnesota, United States

## Abstract

Restless legs syndrome (RLS), a common neurologic disorder, is more prevalent in women than in men. Evidence indicates the potential role of female hormones in the pathophysiology of RLS. However, few studies have examined the relationship between premenopausal bilateral oophorectomy (BSO) and the risk of RLS. The Mayo Clinic Cohort Study of Oophorectomy and Aging-2 (MOA-2) includes all women residents of Olmsted County, Minnesota, who underwent premenopausal BSO before the age of 50 years for a non-cancer reason between 1988 and 2007 (n=1,653). In addition, 1,653 age-matched (± 1 year) referent women who did not undergo BSO prior to index-date were included. Survival analyses were used to examine the association between BSO and risk of RLS (defined by DSM-IV criteria) after adjustments using inverse probability weighting. The median (IQR) age at index-date was 40.0 (40.0-47.0) years for the BSO and 40.0 (40.0-47.0) for the non-BSO cohorts. Women who underwent BSO before menopause had a higher risk of RLS (HR,1.47; 95% CI, 1.08-2.01). In addition, women who underwent BSO before 46 years (HR:1.53, 95% CI, 1.03-2.28) or received BSO without benign ovarian indications (e.g., cysts, endometriosis, HR: 1.69, 95% CI, 1.11-2.56) were at greater risk of RLS. Estrogen use after BSO did not affect the risk of RLS. Premenopausal women who underwent BSO had an increased risk of RLS, especially those younger than 46 years and without benign medical indication. Women considering BSO for ovarian cancer prevention should be informed of the increased risk of neurological disorder including RLS.

